# Comparative maturation of cynomolgus monkey oocytes *in vivo *and *in vitro*

**DOI:** 10.1186/1477-7827-4-14

**Published:** 2006-04-04

**Authors:** Hang Yin, Diane M Duffy, Roger G Gosden

**Affiliations:** 1 The Jones Institute for Reproductive Medicine, Department of Obstetrics and Gynaecology Eastern Virginia Medical School, Norfolk, VA 23507, USA; 2 Department of Physiological Sciences, Eastern Virginia Medical School, Norfolk, VA 23507, USA

## Abstract

**Background:**

In vitro maturation (IVM) of oocytes followed by fertilization in vitro (IVF) and embryo transfer offers an alternative to conventional IVF treatment that minimises drug administration and avoids ovarian hyperstimulation. However, the technique is less efficient than maturation in vivo. In the present study, a non-human primate model was used to address the hypothesis that the number of oocytes is increased and their nuclear and cytoplasmic maturity after IVM are improved when maturation is initiated in vivo by priming with hCG.

**Methods:**

Young, adult cynomolgus monkeys were given recombinant human (rh) gonadotropins to stimulate the development of multiple follicles, and oocytes were aspirated 0, 12, 24, or 36 h after injection of an ovulatory dose of rhCG. The nuclear status of oocytes was determined at the time of recovery and after culture for a total elapsed time of 40–44 hours after hCG.

**Results:**

Priming with hCG significantly increased the number of oocytes harvested, especially after delaying aspiration for 24 h or longer. Nuclear maturation after the full period in culture was also enhanced by priming: 71.5, 83.6, and 94.6% of oocytes collected at 0, 12, and 24 h hCG had progressed to MII by the end of the culture period, compared to 87.8% of oocytes that were retrieved at 36 h. A large proportion of oocytes reaching the MII stage had either or both abnormal spindles (>40%) and misaligned chromosomes (>60%), judging by immunofluorescence microscopy, but these abnormalities were independent of culture time. The mitochondria were evenly distributed throughout the cytoplasm at all stages of maturation. Importantly, there was no microscopic evidence that the duration of culture had any injurious effects on the cells.

**Conclusion:**

In conclusion, the evidence supports this non-human primate as a model for human IVM and the practice of priming with hCG to promote developmental potential.

## Background

In vitro maturation (IVM) is a culture technology that enables a high proportion of fully-grown oocytes at the germinal vesicle (GV) stage to reach metaphase II (MII). When mature, the oocytes may be fertilised in vitro and they become available for transfer to a physiologically synchronised reproductive tract after reaching the cleavage or blastocyst stage. While pregnancy rates after IVM can be impressively high in some laboratory and farm animal species [1-6], applications for human reproductive medicine are just now gathering momentum.

The first human birth reported after IVM was achieved after controlled ovarian stimulation [7]. More than two decades later, several hundred live births have resulted from IVM-IVF, often in cycles lacking FSH stimulation rather than rescuing immature gametes after conventional ovarian stimulation, as in the case above [8]. There is still no standard protocol for IVM, but protocols normally avoid the use of FSH in the interests of simplifying treatment, reducing drug administration and avoiding ovarian hyperstimulation syndrome in women with polycystic ovaries [9]. A single injection of 10,000 IU hCG has been advocated for initiating maturation in vivo before transferring oocytes to culture, because it increased the number and quality of the gametes obtained [10-12]. Pregnancy and implantation rates for IVM-IVF are, however, generally lower than after conventional IVF treatment in the same centers, and the incidence of miscarriage is usually higher. This experience has encouraged the practice of transferring on average slightly more embryos to compensate for the lower pregnancy rates, but this unfortunately raises the risk of multiple pregnancy. Optimal protocols for recovery and culture are needed to increase the success of IVM with the ultimate aim of single embryo transfer.

Progress with IVM has been hindered by the scarcity of suitable human oocytes for research. A non-human primate model is highly desirable because the physiology of the menstrual cycle and embryology are more comparable to humans than other model species. Schramm & Bavister [13] reported the first simian blastocysts created after IVM, and four years later the first live-born infant was announced by the same center [14]. Several culture media have been used in humans and monkeys, and the rates of oocyte maturation to MII have been in the range 60–80% in most reports [7,15-20]. More disappointingly, the rates of blastocyst formation and pregnancy were only 10–40% and 15–30%, respectively, implying that developmental competence was impaired [17-19,21]. It was not clear whether the excess reproductive wastage was due to chromosomal aberrations, which are common in human oocytes. The ability to undergo nuclear maturation is necessary but not sufficient for fertility, which requires faithful congression of chromosomes on the spindle equator and other less well-understood changes that have been collectively called "cytoplasmic maturation". Nevertheless, it is evident that culture conditions are not yet able to faithfully mimic the intrafollicular environment [22].

Accordingly, oocytes that initiated maturation in vivo before transfer to culture should produce better quality gametes. There is bi-directional flow of signals between oocytes and granulosa cells via trans-zonal projections and the extracellular fluid [23]. Cellular stress resulting from manipulation, changing environments and mechanical injury to delicate processes (especially during the early hours of maturation) could impair the balanced coordination of nuclear and cytoplasmic maturation [24]. We further propose that the aforementioned benefit of hCG priming of human ovaries is due to the initiation of physiological events associated with a critical period of maturation before oocytes are exposed to sub-optimal conditions in vitro. This hypothesis was tested by collecting cynomolgus monkey oocytes before and at specific times up to 36 h after hCG treatment. In an ideal model, the quality of oocytes would be as susceptible as in humans to errors generated during meiosis [25,26]. Although rarely studied, cytogenetic errors have been reported in monkey oocytes, supporting their value as a model for human reproductive biology and technology [27]. The integrity of the spindle apparatus and the distribution of mitochondria have been studied here, because they provide indications of oocyte competence after exposure to culture conditions [28-31].

## Materials and methods

### Animals

Adult female cynomolgus monkeys (*Macaca fascicularis*) aged 8.5 ± 0.3 years were studied under the approval of the Institutional Animal Care and Use Committee at the Eastern Virginia Medical School and in accordance with the NIH Guide for the Care and Use of Laboratory Animals. Monkey chow (Agway, Elizabeth City, NJ) was provided twice per day and water was available *ad libitum*. Animals were maintained as social pairs with a light:dark cycle of 12:12 h at 23°C. The animals had regular menstrual cycles and were inspected daily for signs of the onset of menstruation.

### Ovarian stimulation and oocyte collection

A total of 39 ovarian stimulation cycles from 18 animals was performed. Each animal was stimulated from 1 to 3 cycles with at least two months intervals. A standard protocol for controlled ovarian stimulation was used to obtain multiple oocytes [32]. Blood samples were collected by femoral venepuncture under ketamine chemical restraint. Stimulation started within 1–3 days after initiation of menses by administering 60IU recombinant-human FSH i.m. (r-hFSH, Serono Reproductive Biology Institute, Rockland, MA) twice daily for 6–8 days, followed by 60 IU r-hFSH and 45 IU r-hLH twice daily for 2 days to stimulate multiple follicle growth. The GnRH antagonist, Antide, was administered daily (0.5 mg/kg body weight in propylene glycol: water (1:1); Serono) to prevent an endogenous LH surge. Follicle development was monitored by serum oestradiol concentrations and ultrasonography. Follicle aspiration was performed during aseptic surgery with a syringe and a 23 g needle by either laparotomy or laparoscopy on anaesthetised monkeys either without hCG priming (recorded as 0 h) or 12, 24, or 36 h after receiving 1000 IU r-hCG i. m. [33]. In the laboratory, follicular aspirates were diluted in Talp-Hepes medium containing 0.3% bovine serum albumin (BSA), and oocytes were mechanically removed with the aid of a dissecting microscope. Only oocytes that appeared morphologically normal and surrounded by cumulus cells were studied in culture.

### Oocyte culture

The oocytes were cultured in 50 μl droplets of M-199 medium (Gibco Cell Culture, Invitrogen, Carlsbad, CA) under a layer of embryo-tested mineral oil (Sigma) at 37°C in an atmosphere of 5% CO_2 _and 95% air for a total 40–44 h post-hCG. This time was chosen because oocytes are routinely retrieved 36 h after hCG in patients, and IVF or (more typically) ICSI is performed at least 4 h afterwards. The medium was supplemented with 10% fetal bovine serum (Hyclone, Logan, UT), 1 μg/ml oestradiol-17β (Sigma) and 75 μIU/ml of both r-hFSH and r-hLH (Serono). The stage of maturation was evaluated under an inverted phase contrast microscope based on the degree of cumulus expansion and emission of the first polar body, confirming that oocytes had reached MII. Oocytes with a GV nucleus were assumed to be still at prophase I, and those with neither a nucleus nor a polar body were classified as GVBD (germinal vesicle breakdown) and presumed to be at the metaphase I (MI) stage. At the time of retrieval, very few oocytes appeared dark and/or contracted (presumably degenerated) or contained pronuclei or had cleaved spontaneously.

### Immunocytochemistry of spindles and mitochondria

The cumulus cells were removed after brief exposure to 30 IU/ml hyaluronidase (Sigma) in M-199 medium under oil. For spindle analysis, oocytes were fixed according to a protocol modified from Baka et al. [34]. Oocytes were fixed in microtubule-stabilising buffer containing 3.7% formaldehyde and 0.5% Triton X-100 (Sigma) at 37°C for 30 min. To reduce background cytoplasmic staining, fixed oocytes were incubated in a blocking solution overnight at 4°C, which containing 2% BSA (Sigma), 2% Carnation powdered skim milk (Nestle USA, Glendale, CA), 2% normal rabbit serum (Sigma), 0.1 M glycine (Sigma) and 0.01% Triton X-100 in PBS. For spindle staining, oocytes were incubated for 1 h at 37°C with a mouse monoclonal anti-α-tubulin antibody (Sigma) diluted 1:100 in PBS containing 0.1% BSA and 0.02% sodium azide (Sigma). They were then incubated in blocking solution for another hour. To visualise spindles, oocytes were stained with rabbit anti-mouse immunoglobulin (IgG) conjugated with fluorescein (FITC; Sigma). Chromosomes were stained with 10 μg/ml 4',6'-diamidino-2-phenylindole (DAPI; Sigma) in PBS for 15 min at room temperature. After mounting in glycerol:PBS (9:1, v:v) containing 100 mg/ml of 1,4-diazabicyclo(2.2.2)-octane (DABCO; Sigma), the antifading reagent, they were examined with a Nikon fluorescence microscope.

To examine the distribution of mitochondria, oocytes were fixed in 3.7% formaldehyde for 20 min at room temperature after removing cumulus cells using hyaluronidase (30 IU/ml). They were stained in 140 nM MitoTracker® Red 580 (Molecular Probes, Eugene, OR) for 20 min at room temperature. The DNA was stained with DAPI. Oocytes were mounted on slides as described above, and the distribution of mitochondria was analysed by standard fluorescence and confocal microscopy.

### Statistical analysis

Comparisons between groups of oocytes were performed using Chi-square, Fisher's exact test and ANOVA with Tukey-Kramer Multiple Comparison Test as a post test. Results were considered significantly different if p ≤ 0.05.

## Results

A total of 424 oocytes were retrieved from 18 animals, with a mean number of oocytes at 10.9 per animal and per cycle. The numbers of oocytes recovered increased significantly with time elapsed after the injection of hCG (Table 1). At retrieval, the developmental stage of the oocytes could be clearly defined by the presence or absence of the GV nucleus and/or polar body. The large majority in the group that did not receive hCG priming (i.e. 0 h) remained at the GV stage (93.2%). The proportions at the GV stage declined significantly as the interval between hCG and oocyte recovery increased, with a corresponding rise in those progressing to the GVBD stage (Table 1). Between 12–24 h post-hCG, more than half of the oocytes were scored as GVBD. Only 2.3% of the oocytes (7/301) had reached the MII stage before 36 h, and these were likely to have been in atretic follicles. However, by 36 h after hCG priming, the majority of oocytes (87.8%) were MII at aspiration and presumably ripe for fertilization.

**Table 1:** Number and developmental stage of oocytes at retrieval at different times after hCG injection.

Time of oocyte retrieval post-hCG (time in culture) (h)	Number of oocyes	Oocytes per cycle*	GV(%)	GVBD(%)	MII(%)
0(40–44)	147	8.2 ± 0.8	137(93.2)	5(3.4)	5(3.4)
12(38–42)	74	9.3 ± 1.1	61(82.4)	11(14.9)	2(2.7)
24(16–20)	80	13.3 ± 3.8	6(7.5)	74(92.5)	0 (0)
36(4–8)	123	17.6 ± 2.3**	4(3.3)	11(8.9)	108(87.8)

GV: germinal vesicle, GVBD: germinal vesicle breakdown, MII: metaphase II *Mean ± SEM. ANOVA was performed, and **: P < 0.01 compared with group at 0 h, P < 0.05 compared with the group at 12 h

After transfer to culture, most GV and GVBD oocytes resumed maturation towards the MII stage (Table 2). The smallest percentage reaching MII was in the group that was cultured for longest (0 h post-hCG), i.e., only 98/137 (71.5%) reached MII. In the 12 h post-hCG group, which were in culture for a correspondingly shorter time, there were initially 61 GV oocytes, of which 55 underwent GVBD during the subsequent 28–32 h in culture; and 51 of total (83.6%) finally reached MII. A majority of oocytes collected 24 h post-hCG were at the GVBD stage and went forward during the 16–20 h culture period to the MII stage (94.6%). Oocytes recovered from follicles 36 h post-hCG had 87.8% (108/123) reached the MII stage without undergoing parthenogenetic activation or showing any obvious signs of degeneration. On the other hand, in total of five oocytes and another four that were cultured for longer period underwent these respective changes (Table 2). It was unlikely that by extending the culture period for a few hours the proportion of mature oocytes would have increased in either the 0 or 12 h post-hCG groups, because most immature gametes were still at the GV stage (Table 2). All five oocytes retrieved at the GVBD stage without the benefit of hCG priming, and 10 of 11 GVBD oocytes collected at 12 h completed maturation after transfer to culture. But only one of the six GV oocytes collected at 24 h post-hCG developed to GVBD, and the rest remained arrested. Among the GV oocytes collected 36 h post-hCG and cultured for 4–8 h, only 1 of 4 underwent GVBD, and 4 of 11 at GVBD arrested at anaphase I. It appeared, therefore, that there is little to gain by culturing oocytes after hCG priming, either because the oocytes are meiotically incompetent or culture conditions are suboptimal. Overall, the data indicated that the longer the period of maturation in vivo (or the shorter the culture period) the greater the harvest of mature gametes. Nevertheless, a respectable proportion of >70% of GV oocytes reached nuclear maturity, even in the group cultured for the maximum time.

**Table 2:** Number of oocytes resuming development in culture and analysed 40–44 h after hCG priming and variable times in culture

	Stage of Oocytes after Culture						
Time of oocyte retrieval post-hCG (time in culture) (h)	Number of oocytes (stage at initiation of culture)	GV(%)	GVBD (%)	Anaphase I (%)	MII (%)		
0(40–44)	137(GV)	13(9.5)	10(7.3)	1(0.7)	98(71.5)	4(2.3)	3(2.2)
12(28–32)	61(GV)	6(9.8)	2(3.3)	1(1.6)	51(83.6)*	-	1(1.6)
24(16–20)	74(GVBD)	-	3(4.1)	-	70(94.6)**	1(1.3)	-
36(4–8)	108(MII)		-	-	-	-	-

Fisher's exact test was performed. *P < 0.05; and **P < 0.001 compared with 0 h group

**Figure 1:**
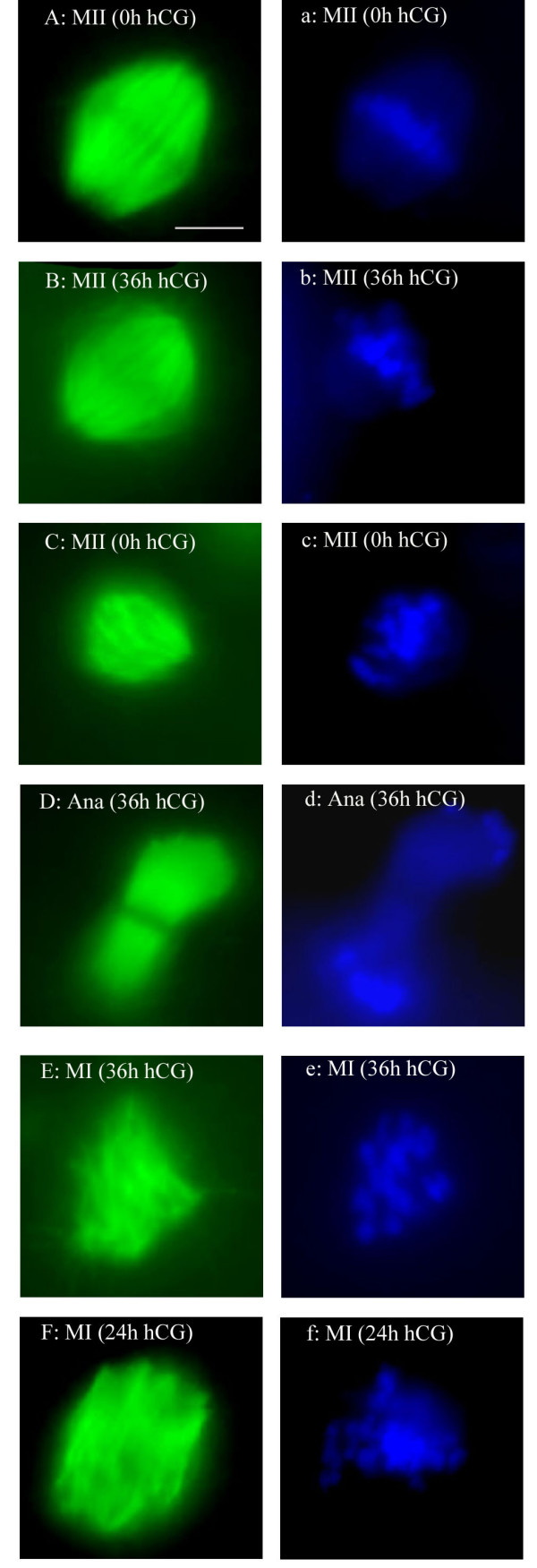
Spindle formation and chromosome alignment in oocytes. A, B, C, D, E and F showing spindles in green after staining with an anti α-tubulin antibody, and a, b, c, d, e and f showing chromosomes in blue after DAPI staining (chromatin). Normal bi-polar spindle and well-aligned chromosomes were present in MII oocytes (0 h) after 40–44 h entirely in culture (A and a), or in an oocyte 36 h post-hCG (B and b); asymmetric spindle and displaced chromosomes around the spindle equator plate in a MII oocyte after 40–44 h in culture (C and c); an oocyte retrieved at 36 h post-hCG was arrested at anaphase I (D and d); MI oocyte retrieved 36 h post-hCG possessed abnormal spindles and disorganised chromosomes (E and e); a cultured oocyte retrieved at 24 h post-hCG was arrested at GVBD with an abnormal spindle and disorganised chromosomes (F and f). Magnification bar in (A) = 5 μm

Normally, MII monkey oocytes have a bipolar, barrel-shaped spindle with chromosomes aligned uniformly at the equator plate (Figure 1A, a). Various abnormal forms were observed in this study, including asymmetric, tripolar and depolymerised spindles, as well as displaced, lagging and disorganised chromosomes. There were more oocytes with abnormal chromosome alignments than abnormal spindles, and only 23% (65/280) of oocytes were normal in both respects. The high incidence of anomalies was striking: less than half of the oocytes had a normal spindle and only a third had well-aligned chromosomes. There were apparently fewer normal spindles at 24 h, and the difference was marginally significant compared to the data at 0 h (p = 0.040) and 12 h (p = 0.015, Table 3). None of the oocytes arrested at the GVBD stage after culture in any of the experimental groups had a normal MI spindle and chromosome congression at the equator plate (Figure 1F, f). There were four oocytes with abnormal, tripolar spindles, which may be an indication of defective cellular polarity.

**Table 3:** Spindle structure and chromosome alignment in MII oocytes analysed 40–44 h after hCG priming and variable times in culture

Time of oocyte retrieval post-hCG (time in culture) (h)	Number of oocytes	Spindle structure (%)		Chromosome alignment (%)	
		
		Normal	Abnormal	Normal	Abnormal
0(40–44)	89	44 (49.4)	45 (50.6)	30 (33.7)	59 (66.3)
12(28–32)	44	25 (56.8)	19 (43.2)	15 (34.1)	29 (65.9)
24(16–20)	58	18 (31.0)	40 (69.0)*	21 (36.2)	37 (63.8)
36(4–8)	89	40 (44.9)	49 (55.1)	28 (31.5)	61 (68.5)

Fisher's exact test was performed. * P < 0.05 compared with groups at 0 and 12 h

**Figure 2:**
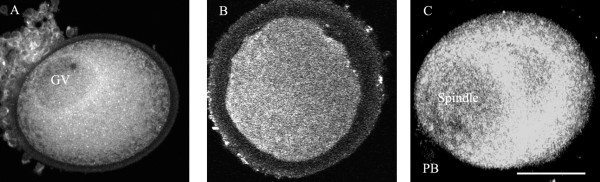
The distribution of mitochondria in oocytes after 40–44 h in culture (0 h group) and staining with the mitochondrial dye, MitoTracker^® ^Red 580. A. Mitochondria surrounding the nucleus (GV) in a freshly-retrieved oocyte. B. Oocyte still arrested at GVBD with mitochondria evenly distributed in cytoplasm. C. Oocyte at MII with mitochondria congregated around the region of spindle and in cytoplasm. Magnification bar in (C) = 50 μm.

A total of 99 oocytes at GV, GVBD and MII stages were stained for mitochondria (Table 4). These organelles were evenly distributed throughout the cytoplasm of the cells. There was no obvious congregation of mitochondria around the GV nucleus or the spindle of MI and MII oocytes (Figure 2), and their distribution did not differ between GV and MII stages or in relation to time in culture.

**Table 4:** Number and developmental stage of oocytes stained to reveal the distribution of mitochondria 40–44 h after priming with hCG and variable times in culture

Time of oocyte retrieval post-hCG (time in culture) (h)	Number of oocytes	GV	GVBD	MII
0(40–44)	29	10	5	14
12(28–32)	25	6	2	17
24(16–20)	18	4	2	12
36(4–8)	27	3	6	18

## Discussion

This study has demonstrated that the timing of oocyte collection after stimulating cynomolgus monkey ovaries with hCG has a striking effect on the prospects for IVM. The main effect was on the numbers of oocytes recovered, a larger harvest being obtained when the collection was delayed after hCG priming. This result can be accounted for by a lower efficiency of aspirating immature oocytes with unexpanded cumulus masses. The efficiency of collection from unstimulated human ovaries has been improved by curetting follicles with a modified needle and a lower aspiration pressure [15]; technical modifications may also improve oocyte harvests in unstimulated monkey ovaries.

These data confirmed that the time taken for monkey oocytes to reach MI and MII are comparable to humans [35-37]. More than 12 h was required to undergo GVBD, compared with only 2–4 h in the mouse, and MII was not reached until 36 h, compared to 12–16 h [38,39]. The data indicated that the proportion of oocytes becoming mature was significantly increased after hCG priming (Table 2). Without continuous observation in vitro, it was not possible to assess variations between gametes in the time taken to mature, which ranges from 24–48 h for human oocytes [15]. Priming with hCG accelerates the maturation of human oocytes in vitro [40,12], but this finding was based on patients with unstimulated polycystic ovaries, and it is not known if this applies to normal ovareis. Overall, human and simian studies appear to be concordant insofar that they reveal that hCG treatment can be beneficial, although the end-points were different.

The number of oocytes available for fertilization and transfer is a key predictor of pregnancy rates after IVM in humans [41]. Although fertilization and progression to the blastocyst stage were not addressed in the present study, our results revealed that a longer post-hCG interval decreased the percentage still at the GV stage, and only 3.3% were still arrested at 36 h. The intrafollicular environment is evidently more effective at supporting maturation than culture conditions that were based on a formula widely used in humans and domestic animals [42-44]. There are several formulas for human IVM but, with few exception [16], they all contain serum, and efforts to produce an optimal medium, and preferably chemically-defined, have been thwarted by the lack of research material. Culture medium supplemented with steroids and growth factors, including oestradiol, progesterone, EGF, IGF-1 and VEGF, have been reported to improve maturation and developmental competence in various animal species [45-50]. However, optimal conditions probably vary between species and even at different phases of the 36 h incubation period, reflecting the evolving physiological state of the preovulatory follicle. The challenge of improving the clinical success rate with IVM is most acute when oocytes are most limiting, as in the case of natural cycles [51] and cancer patients for whom only one harvest may be feasible [52]. Moreover, that challenge would be also beneficial in the case of somatic cell nuclear replacement, especially in non-human primates and human.

Human oocytes often have small, aberrant spindles with unaligned chromosomes whose frequency rises with maternal age [53,54]. Since they are prone to meiotic errors [54], it has been suggested that oocytes lack a spindle checkpoint to control the fidelity of the metaphase/anaphase transition during the first meiotic division [55]. A remarkably high frequency of abnormal spindles and displaced chromosomes was found in the present study, even though the monkeys were only about eight years old, which is long before reproductive senescence in this species [27]. The high association between spindle integrity and chromosome misalignment indicated that spindle morphology is predictive of aneuploidy, as has been demonstrated in rodent models [30], and it is likely that the same relationship holds in primates. Overall, such findings suggest that spindle defects compromise the quality of monkey oocytes, it is important to note that the incidence was not increased with duration of culture in the present study. However, the effects of ovarian stimulation regimes, such as GnRH, FSH, LH and hCG, on spindle formation and chromosome alignment in non-human primate oocytes may be a focus in the future study.

Mitochondria play an essential role in oocyte and embryo metabolism [31]. Spindle assembly and function are likely to be impaired if ATP production is impaired locally or globally within the cell [30], which could arise from mitochondrial DNA deletions and/or rearrangements [56,57]. No evidence has yet been published to show a harmful effect of culture on mitochondrial DNA during IVM, although changes observed in the cytoskeleton could impact the distribution and, hence, local activity of these organelles [28,58]. The distribution of mitochondria varies over time and between species during oocyte maturation and embryo cleavage [28,59-61]. In the present study, the distribution of mitochondria was even throughout the cytoplasm during the process of maturation, confirming an earlier report [62], and was not affected by time in culture. In view of these findings, it is doubtful if the distribution of these organelles can serve as a useful biomarker for developmental competence of monkey oocytes.

Overall, this study affirms the value of the non-human primate model for optimizing IVM protocols in clinical applications. The findings confirmed the hypothesis that initiation of maturation after priming with hCG is beneficial and provided evidence that the high background incidence of potential cytogenetic errors in cynomolgus monkey oocytes is not raised by culture conditions.
